# Synthesis and crystallographic analysis of *meso*-2,3-difluoro-1,4-butanediol and *meso*-1,4-dibenzyloxy-2,3-difluorobutane

**DOI:** 10.3762/bjoc.6.62

**Published:** 2010-06-08

**Authors:** Bruno Linclau, Leo Leung, Jean Nonnenmacher, Graham Tizzard

**Affiliations:** 1 School of Chemistry, University of Southampton, Highfield, Southampton SO17 1BJ, UK

**Keywords:** building block, epoxide opening, gauche effect, organofluorine, vicinal difluoride

## Abstract

A large-scale synthesis of *meso*-2,3-difluoro-1,4-butanediol in 5 steps from (*Z*)-but-2-enediol is described. Crystallographic analysis of the diol and the corresponding benzyl ether reveals an *anti* conformation of the vicinal difluoride moiety. Monosilylation of the diol is high-yielding but all attempts to achieve chain extension through addition of alkyl Grignard and acetylide nucleophiles failed.

## Introduction

Selective fluorination of bioactive compounds is a widely employed strategy for the modification of their properties [1]. Fluorine atoms can be introduced to modulate the p*K*_a_ of adjacent acidic and basic functional groups as well as the lipophilicity, chemical and metabolic stability of the compound. Recent exciting reports describe weak but stabilising interactions between a C–F moiety and protein residues, which is certain to have implications in drug design [2–3]. Further important applications include molecular imaging using ^18^F [4], and modification of high-performance materials [5].

In recent years, the vicinal difluoride motif has received increasing attention due to the conformational properties instilled by the ‘gauche effect’ [6], which results in the vicinal difluoro *gauche* conformation being more stable than the corresponding *anti* conformation [7–9]. O’Hagan has demonstrated that vicinal difluoride substitution along a hydrocarbon chain of a fatty acid leads to conformational rigidity or disorder depending on the relative stereochemistry of the fluorine atoms, which originates from the enforcing or opposing fluorine *gauche* and hydrocarbon *anti* low-energy conformations [10]. As an extension, multi-vicinal tri- to hexafluorinated chains have been synthesised [11–16], which revealed yet another effect on the conformational behaviour, i.e. that conformations containing parallel 1,3-C–F bonds are destabilised. As an application, liquid crystals have been prepared containing a vicinal difluoride motif [14,17–18].

Efficient stereodefined synthesis of vicinal difluoride moieties is not straightforward. Direct methods include fluorination of alkenes with F_2_ [19], XeF_2_ [20], or hypervalent iodine species [21]. Such approaches often display poor stereoselectivity or result in rearrangement products. Treatment of 1,2-diols with SF_4_ [22–23], DAST [24], or deoxofluor [25] also leads to vicinal difluorides. Reaction with vicinal triflates has also been successful in some cases [7,26]. A common two-step method involves opening of an epoxide to give the corresponding fluorohydrin [27], followed by the conversion of the alcohol moiety to the fluoride [28]. Another two-step method is halofluorination of alkenes and subsequent halide substitution with silver fluoride [9,29–30].

**Figure 1:**
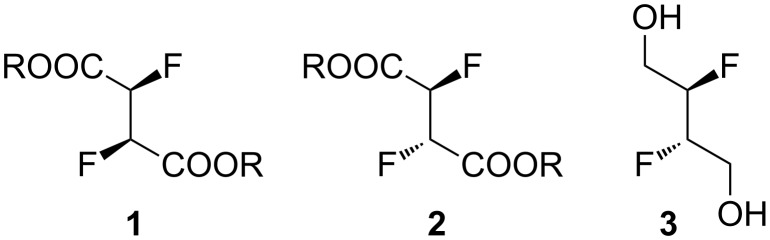
Vicinal difluoride containing building blocks.

The introduction of multiple fluorine atoms is often a cumbersome process, and in many cases a fluorinated building block approach [31–32] is more efficient. Known vicinal difluoride containing building blocks include (racemic) *C*_2_-symmetric and *meso*-2,3-difluorosuccinic acids (or esters) **1**,**2** (Figure 1) [9,22–23,33–34].

Herein we describe the first synthesis of *meso*-2,3-difluoro-1,4-butanediol **3** as a further simple vicinal difluoride building block as well as its successful monosilylation, and our attempts to employ **3** for the synthesis of fluorinated hydrocarbons.

## Results and Discussion

### Synthesis

The synthesis of **3** was achieved from *meso*-epoxide **4**, which was obtained from (*Z*)-2-butene-1,4-diol in excellent yield according to the published two-step sequence [35]. The optimisation of the reaction of **4** with fluoride sources is shown in Table 1.

**Table 1:** Conversion of epoxide **4** to the fluorohydrin.

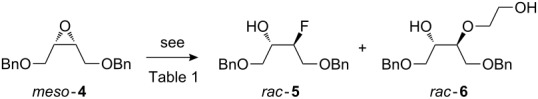				

Entry	Reaction conditions	**5**^a^	**6**^a^	**4**^a^

1	HF•py (70% HF), r.t., 3 h	80^b^	–	–
2	KHF_2_, ethylene glycol, 150 °C, 3 h	34	50	–
3	KHF_2_, ethylene glycol, mol. Sieves, 150 °C, 3 h	–	^c^	–
4	KHF_2_, DMSO, 150 °C, 16 h	–	–	^d^
5	KHF_2_, DMF, 18-crown-6, reflux, 16 h	–	–	^d^
6	Bu_4_NH_2_F_3_ (1 equiv), xylene, reflux, 3 d	11	–	57
7	Bu_4_NH_2_F_3_ (1 equiv), KHF_2_ (1 equiv), 130 °C, 16 h	71	–	–
8	Bu_4_NH_2_F_3_ (1 equiv), KHF_2_ (1 equiv), 115 °C, 2.5 d	91	–	–

^a^ Isolated yield. ^b^ Mixture of isomers. ^c^ Complete conversion to **6** (TLC analysis). ^d^ No reaction observed.

Reaction with Olah’s reagent [29] proceeded in excellent yield (Table 1, entry 1), however, the product was isolated as a mixture of isomers, which were not further characterised. Reaction with potassium hydrogen difluoride in ethylene glycol [36–37] gave the fluorohydrin in only modest yield (entry 2). Interestingly, the product arising from epoxide ring opening by ethylene glycol, **6**, was isolated in 50% yield. The addition of molecular sieves (entry 3) led to complete conversion to **6** (TLC analysis). No reaction took place when DMSO (entry 4) or DMF/18-crown-6 were used as solvents [38–39] (entry 5). With Bu_4_NH_2_F_3_ as the fluoride source [40–41], 11% of the desired product (together with some elimination byproducts) was obtained when xylene was used as solvent (entry 6). However, reaction with a mixture of Bu_4_NH_2_F_3_ and KHF_2_ in the absence of solvent [42–44] led to an excellent 91% yield of the desired product **5** albeit after a relatively long reaction time (entry 8).

**Scheme 1:**
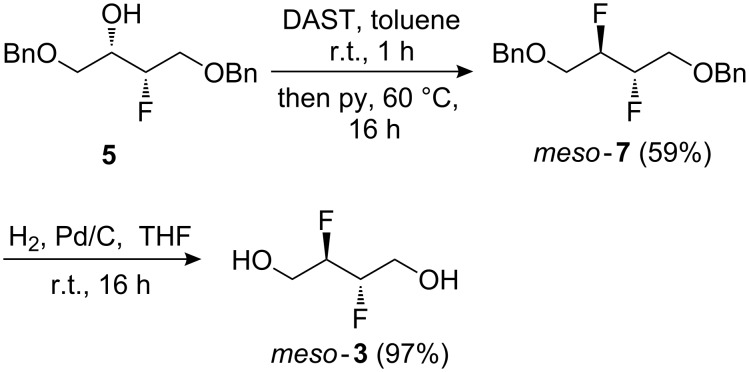
Synthesis of *meso*-2,3-difluoro-1,4-butanediol.

The subsequent conversion to **3** is shown in Scheme 1. Treatment of **5** with DAST in DCM at reflux temperature only gave **7** in 29% yield (not shown). A slight improvement (40% yield) was obtained when the reaction was conducted in hexane or toluene, but a procedure in which DAST was added to a solution of **5** in toluene at room temperature, followed by the addition of pyridine [28] and heating the reaction mixture for a prolonged period gave the desired vicinal difluoride in good yield. Nevertheless, while this procedure was deemed sufficiently safe to conduct at about the 50 mmol scale, further upscaling with a more thermally stable fluorinating reagent such as deoxofluor [45], Fluolead [46], or aminodifluorosulfinium tetrafluoroborate [47] would be recommended. Subsequent alcohol deprotection gave the target compound in almost quantitative yield in multigram quantities.

**Scheme 2:**
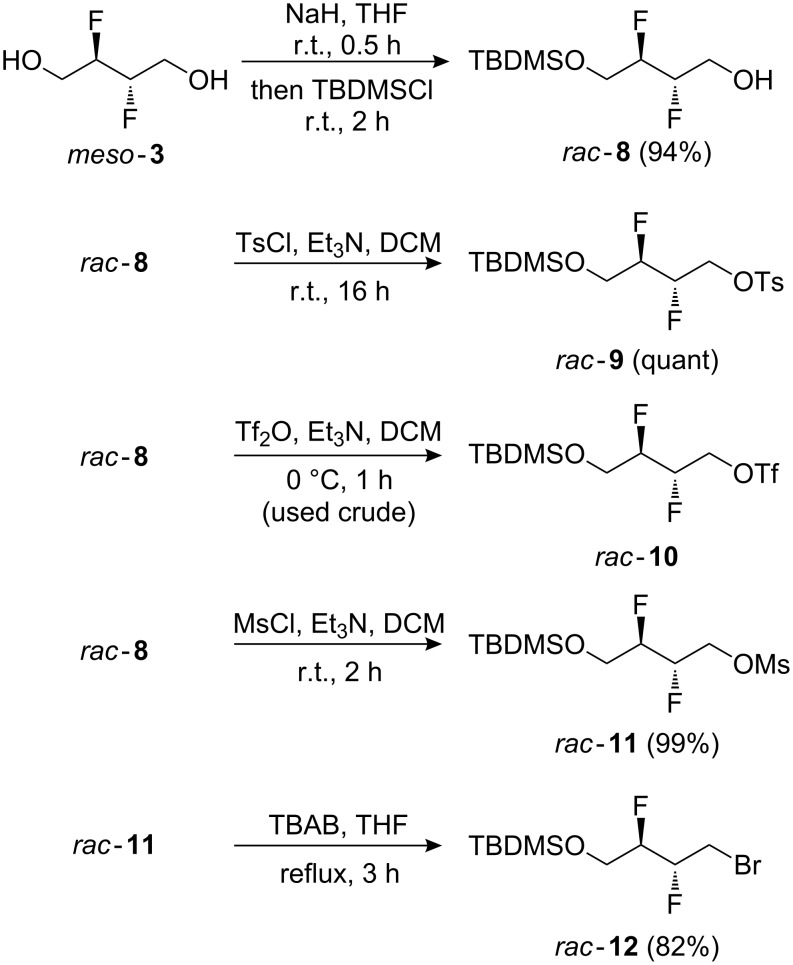
Monoprotection of **3**, and activation of the remaining alcohol.

The potential of **3** as a building block, in particular for the construction of longer aliphatic chains of varying length, was investigated next. Thus (Scheme 2), the diol moiety in **3** was monoprotected as a silyl ether, and the remaining alcohol group was activated as the corresponding tosylate **9**, triflate **10**, mesylate **11**, and bromide **12** as precursors for chain extension. Nucleophilic substitution of similar tosylates with phenolate nucleophiles has been previously described [18]. Reaction of **9**–**12** with a number of carbon nucleophiles was investigated.

**Scheme 3:**
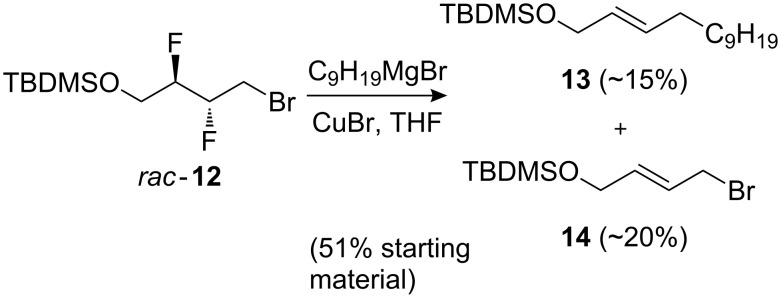
Reaction of **12** leading to defluorinated products.

Unfortunately, reaction of **9**–**12** with alkyl Grignard and acetylide reagents did not lead to the desired chain extension. Reaction of **9** or **10** with a sodium or lithium acetylide led to decomposition, while **12** did not react under these conditions. Treatment of **11** with C_9_H_19_MgBr/CuBr was unsuccessful, whilst surprisingly, when **12** was subjected to this reagent combination (Scheme 3), the defluorinated reaction products **13** and **14** were obtained. We have not yet deduced an acceptable explanation for this unexpected result.

### Crystallographic analysis

**Figure 2:**
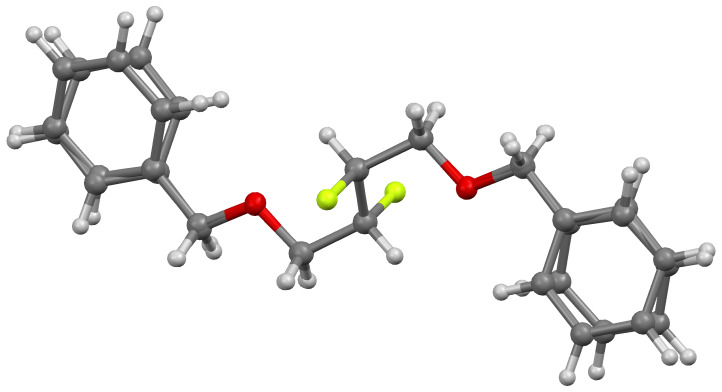
Molecular overlay of both conformers of **7**.

Compounds **7** and **3** yielded colourless crystals suitable for study by single crystal X-ray diffraction [48]. The dibenzyl ether **7** crystallises in the monoclinic *P*2_1_/*c* space group with half a molecule of **7** in the asymmetric unit. The molecule possesses crystallographic inversion symmetry. Two conformers are present in the crystal (55:45) which differ only in the sign of the torsion angle of the rings (Figure 2). The disparity in the amounts of each conformer present gives rise to the disorder observed in the crystal structure.

**Figure 3:**
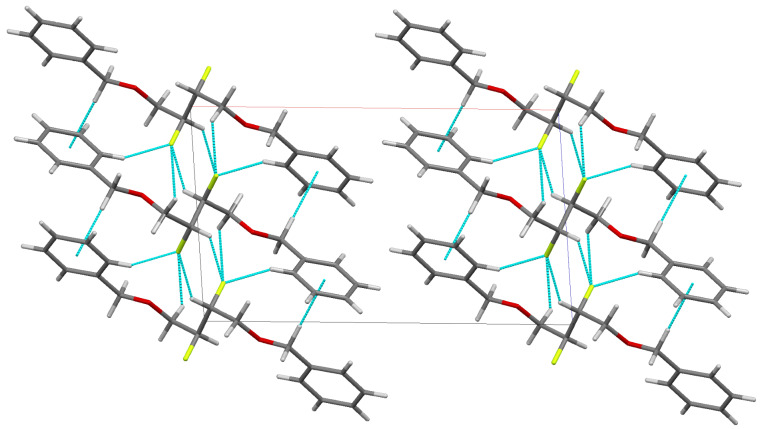
Crystal packing of **7** viewed along the b axis. Short contacts (see text) are shown in light blue.

The vicinal difluoro group adopts an *anti* conformation with the F–C–C–F dihedral angle exactly 180°, which manifests itself in the crystallographic inversion centre. Nevertheless, each benzyloxy group does adopt a *gauche* conformation with its adjacent fluoro substituent where the F–C–C–O dihedral angle is 71.5°. Although strong H-bonding interactions are absent within the crystal, each molecule displays eight short contacts less than the sum of the van der Waals radii to its four nearest neighbours; three C–F···H–C contacts (2.554 Å, 2.581 Å and 2.637 Å) for each fluorine, and a pair of C–H···π contacts (2.662 Å to centroid of ring). The hydrogen atoms involved in the C–F contacts are an aromatic proton, the CHF and a CHHOBn proton (Figure 3).

**Figure 4:**
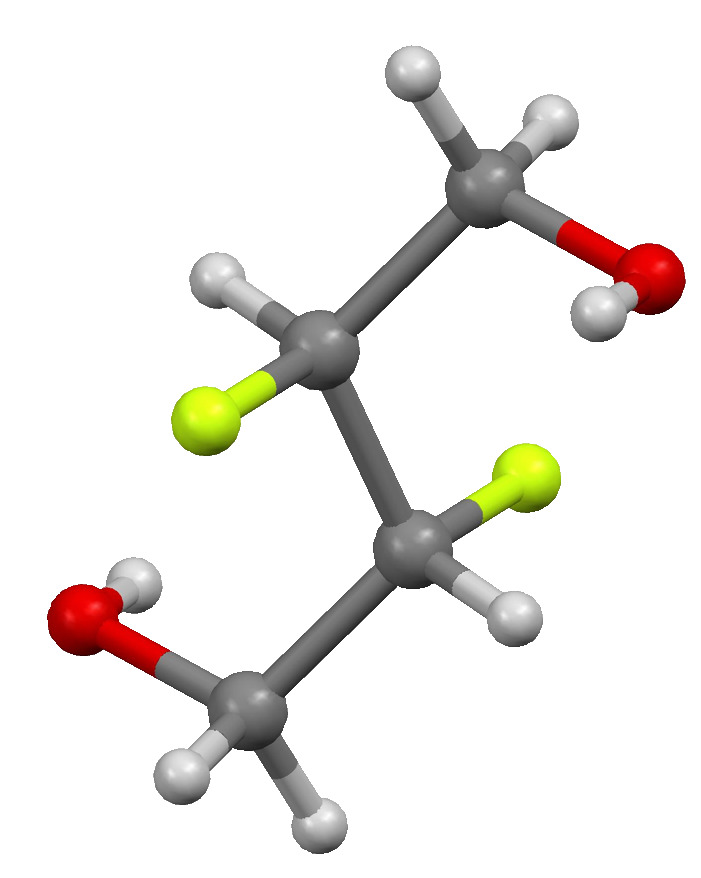
Crystal structure of **3**.

The diol **3** crystallises in the tetragonal space group *I*4_1_/*a* with half a molecule of **3** in the asymmetric unit. This molecule also displays crystallographic inversion symmetry. In common with **7**, the vicinal difluoro group of **3** adopts an *anti* conformation with a symmetry-constrained dihedral angle of 180°, and the hydroxyl groups adopt *gauche* conformations with the adjacent fluoro atoms with F–C–C–O dihedral angles of 66.8° (Figure 4).

**Figure 5:**
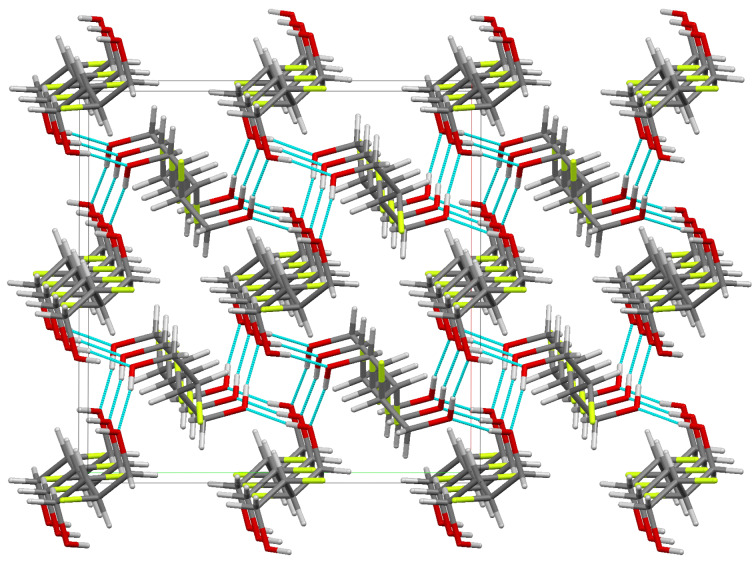
Crystal packing of **3** viewed along the c axis. H-bonds are shown in light blue.

There is strong hydrogen bonding between the hydroxyl groups of the molecule with each hydroxyl group acting both as donor and acceptor (O–H···O: 2.685 Å, 170.1°). The hydrogen bonded molecules are arranged helically about the crystallographic 4_1_ screw axes. Thus the crystal structure comprises of alternating left and right handed hydrogen bonded helical constructs with each molecule part of two adjacent helices (Figure 5).

Examination of the Cambridge Structural Database [49] (V5.31, November 2009) revealed three more *meso*-*vic*-difluoro compounds: 1,2-difluoro-1,2-diphenylethane, 2,3-difluorosuccinic acid and 2,3-difluorosuccinate benzylamide, all reported by O’Hagan [9]. Of these, only difluorosuccinic acid crystallises with the vicinal difluoro group in the expected *gauche* conformation, whilst both other structures, in common with the structures described in this work, contain the vicinal difluoro group in an *anti* conformation. The conformation of vicinal difluorides in solution can also be deduced from NMR studies. Schlosser has reported that the ^3^*J*_H-F_ is around 22 Hz when the fluorines are in the *syn* configuration, because of a preferred *gauche* conformation, and around 14 Hz when in the *anti* configuration, because there is no overall preferred conformation [28]. Unfortunately, we were unable to extract ^3^*J*_H-F_ values from the second order signals in both the ^1^H and ^19^F NMR spectra of **3** and **7**, however, analysis of the coupling constants in **11** revealed two ^3^*J*_H-F_ values of 10.1 and 9.6 Hz (^3^*J*_F-F_ 13.5 Hz). Walba et al. have reported the ^3^*J*_H-F_ values of a very similar *syn*-1-hydroxy-4-aryloxy-2,3-difluorobutane system to be around 22.0 Hz [17]. Hence, this value is indeed much higher than the ^3^*J*_H-F_ values for **11**, from which it can be concluded that the gauche effect in **11** (*anti*) is operating in solution.

## Conclusion

The synthesis of *meso*-2,3-difluoro-1,4-butanediol **3** was achieved in 5 steps from (*Z*)-1,4-butenediol in 40% overall yield on a multigram scale. A high-yielding (94%) monosilylation was also achieved, but all attempts for chain extension met with failure. Crystallographic analysis revealed that the vicinal fluorine atoms in **3** and its dibenzyl ether **7** are in the *anti* conformation.

## Experimental

^1^H and ^13^C NMR spectra were recorded at room temperature on a Bruker DPX400 or AV300 spectrometer as indicated. Low resolution ES mass and EIMS were recorded on a Waters ZMD and Thermoquest TraceMS quadrupole spectrometers, respectively. Infrared spectra were recorded as neat films on a Nicolet Impact 380 ATR spectrometer. Melting points were recorded on a Gallencamp Melting Point Apparatus and are uncorrected.

Column chromatography was performed on 230–400 mesh Matrex silica gel. Preparative HPLC was carried out using a Biorad Biosil D 90-10, 250 × 22 mm column eluting at 20 mL min^−1^, connected to a Kontron 475 refractive index detector. Reactions were monitored by TLC (Merck) with detection by KMnO_4_ or anisaldehyde stains.

Reaction solvents were dried before use as follows: THF and Et_2_O were distilled from sodium/benzophenone; CH_2_Cl_2_ and Et_3_N were distilled from CaH_2_; toluene was distilled from sodium.

X-ray data crystal structure analyses: Suitable crystals were selected and data collected on a Bruker Nonius Kappa CCD Area Detector equipped with a Bruker Nonius FR591 rotating anode (λ(MoKα) = 0.71073 Å) at 120 K driven by COLLECT [50] and processed by DENZO [51] software and corrected for absorption by using SADABS [52]. The structures were determined in SHELXS-97 and refined using SHELXL-97 [53]. All non-hydrogen atoms were refined anisotropically with hydrogen atoms included in idealised positions with thermal parameters riding on those of the parent atom.

### *syn*-1,4-Bis(benzyloxy)-3-fluorobutan-2-ol (**5**)


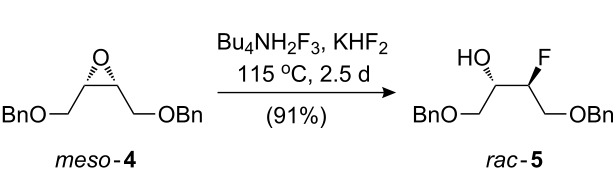


KHF_2_ (9.57g, 123 mmol) was added to a mixture of epoxide **4** (17.4 g, 61.3 mmol) and Bu_4_NH_2_F_3_ (10.6 g, 35.2 mmol) and the mixture stirred at 115 °C for 2.5 days. Et_2_O (300 mL) was added and the solution poured into sat. NaHCO_3_ (200 mL). The organic layer was washed successively with sat. NaHCO_3_ (100 mL) and brine (200 mL), dried over MgSO_4_, filtered and concentrated in vacuo. The crude product was purified by column chromatography (EtOAc/petroleum ether 10% to 20%) to afford fluorohydrin **5** as a colourless oil (17.0 g, 91%). IR *ν*_max_ (cm^−1^) 3062 w, 3030 w, 2993 w, 2858 w, 1496 w, 1453 m, 1369 w, 1088 s; ^1^H NMR (400 MHz, CDCl_3_) 7.42–7.20 (10H, m, ArH), 4.74 (1H, ddt, *J* = 47.5, 5.5, 3.5 Hz, CHF), 4.60 (1H, d, *J* = 12.0 Hz, CH_a_H_b_Ph), 4.58 (1H, d, *J* = 12.0 Hz, CH_c_H_d_Ph), 4.56 (1H, d, *J* = 12.0 Hz, CH_a_H_b_Ph), 4.54 (1H, d, *J* = 12.0 Hz, CH_c_H_d_Ph), 4.04 (1H, dm, *J* = 22.0 Hz, CHOH), 3.80 (1H, ddd, *J* = 23.0, 11.0, 4.0 Hz, CH_a_H_b_OBn), 3.76 (1H, ddd, *J* = 24.0, 11.0, 5.0 Hz, CH_a_H_b_OBn), 3.63 (1H, ddd, *J* = 10.0, 5.0, 1.0 Hz, CH_c_H_d_OBn), 3.59 (1H, ddd, *J* = 10.0, 6.5, 1.0 Hz, CH_c_H_d_OBn), 2.61 (1H, bd, *J* = 4.0 Hz, OH) ppm; ^13^C NMR (100 MHz, CDCl_3_) 137.9 (C_Ar_), 137.7 (C_Ar_), 128.6 (CH_Ar_), 128.0 (CH_Ar_), 127.9 (CH_Ar_), 91.8 (d, *J* = 175.0 Hz, CHF), 73.9 (CH_2_Ph), 73.7 (CH_2_Ph), 70.37 (d, *J* = 5.5 Hz, CH_2_OBn), 70.34 (d, *J* = 20.0 Hz, CHOH), 69.8 (d, *J* = 23.0 Hz, CH_2_OBn) ppm; ^19^F NMR (282 MHz, CDCl_3_) −204.3 (1F, dq, *J* = 46.7, 23.4) ppm; ES^+^ *m*/*z* (%) 327 ((M+Na)^+^, 100); HRMS (ES^+^) for C_18_H_21_FO_3_Na (M+Na)^+^: Calcd 327.1367; Measured 327.1364.

### Data for *syn*-3-(2-hydroxyethyl)-1,4-bis(benzyloxy)butan-2-ol (**6**)


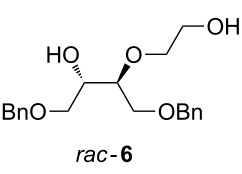


Colourless oil. IR *ν*_max_ (cm^−1^) 3399 br, 3062 w, 3030 w, 2863 w, 1496 w, 1483 m, 1091 s; ^1^H NMR (400 MHz, CDCl_3_) 7.40–7.27 (10H, m), 4.54 (4H, s), 3.87 (1H, q, *J* = 5.5 Hz), 3.78–3.60 (7H, m), 3.58 (1H, dd, *J* = 10.0, 5.0 Hz), 3.51 (1H, dd, *J* = 9.5, 6.0 Hz), 3.25–2.30 (2H, br, OH) ppm; ^13^C NMR (100 MHz, CDCl_3_) 137.9 (C_Ar_), 137.7 (C_Ar_), 128.61 (CH_Ar_), 128.59 (CH_Ar_), 128.01 (CH_Ar_), 127.99 (CH_Ar_), 127.92 (CH_Ar_), 79.4 (CHO), 73.7 (CH_2_Ph), 73.6 (CH_2_Ph), 73.2 (CH_2_O), 71.0 (CH_2_O), 70.9 (CHOH), 70.6 (CH_2_O), 62.3 (CH_2_O) ppm; ES^+^ *m*/*z* (%) 715 ((2M+Na)^+^, 20); HRMS (ES^+^) for C_20_H_26_O_5_Na (M+Na)^+^: Calcd 369.1672; Measured 369.1667.

### *meso*-1,4-Bis(benzyloxy)-2,3-difluorobutane (**7**)


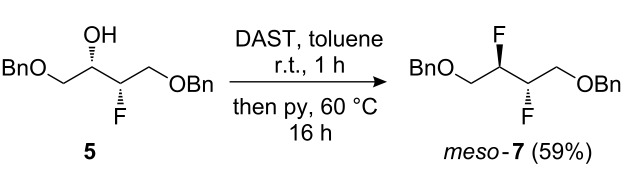


DAST (9.6 mL, 72.7 mmol) was added to a solution of fluorohydrin **5** (17.0 g, 55.9 mmol) in toluene (75 mL) and the mixture stirred at r.t. for 5 min. Pyridine (11.9 mL, 145 mmol) was then added and the solution stirred at 70 °C for a further 16 h. The reaction mixture was cooled, poured into sat. NaHCO_3_ (100 mL) and Et_2_O (100 mL). The organic layer was washed successively with sat. NaHCO_3_ (100 mL) and brine (100 mL), dried over MgSO_4_, filtered and concentrated in vacuo. The crude product was quickly purified by column chromatography (EtOAc/petroleum ether 0% to 5%) to afford a mixture which was recrystallised from hot petroleum ether. The filtrate was concentrated and recrystallised again from hot petroleum ether. The recrystallisation process was carried out for a third time to afford difluoride **7** as a white crystalline solid (overall yield 10.1 g, 59%). mp 56–57 °C; IR *ν*_max_ (cm^−1^) 3058 w, 3030 w, 2916 w, 2878 w, 1607 w, 1496 w, 1449 m, 1137 s, 1048 s; ^1^H NMR (400 MHz, CDCl_3_) 7.40–7.27 (10H, m, ArH), 4.96–4.78 (2H, m, CHF × 2), 4.61 (4H, s, CH_2_Ph × 2), 3.88−3.71 (4H, m, CH_2_OBn) ppm; ^13^C NMR (100 MHz, CDCl_3_) 137.8 (C_Ar_ × 2), 128.6 (CH_Ar_ × 4), 128.0 (CH_Ar_ × 2), 127.8 (CH_Ar_ × 4), 90.0 (dd, *J* = 175.5, 27.5 Hz, ABX, ^13^CHF-^12^CHF × 2), 73.8 (CH_2_Ph × 2), 68.4 (m, ABX, ^13^CH_2_CHFCHF × 2) ppm; ^19^F NMR (282 MHz, CDCl_3_) −198.7 ppm; ES^+^ *m*/*z* (%) 329 ((M+Na)^+^, 100); HRMS (ES^+^) for C_18_H_20_F_2_O_2_Na (M+Na)^+^: Calcd 329.1324; Measured 329.1319.

### *meso*-2,3-Difluorobutane-1,4-diol (**3**)


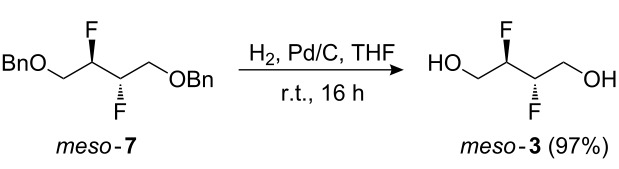


Pd/C (5%; 13.9 g, 6.5 mmol) was added to a solution of difluoride **7** (10.0 g, 32.7 mmol) in THF (108 mL) and the mixture stirred at r.t. for 16 h under a H_2_ atmosphere (balloon). The suspension was filtered through celite, washed with MeOH and concentrated in vacuo. The crude product was purified by column chromatography (acetone/petroleum ether 30% to 50%) to afford diol **3** as a white crystalline solid (4.0 g, 97%). mp 99–101 °C; IR *ν*_max_ (cm^−1^) 3329 br, 2936 br, 1647 br, 1042 s; ^1^H NMR (400 MHz, CDCl_3_) 4.85–4.70 (2H, m, CHF × 2), 4.08–3.83 (4H, m, CH_2_OH × 2), 1.92 (2H, t, *J* = 6.5 Hz, OH × 2) ppm; ^13^C NMR (100 MHz, acetone-*d*_6_) 92.6 (dd, *J* = 173.0, 26.0 Hz, ABX, ^13^CHF-^12^CHF × 2), 61.2 (m, ABX, ^13^CH_2_CHFCHF × 2) ppm; ^19^F{^1^H} NMR (282 MHz, acetone-*d*_6_) −200.5 ppm; HRMS (ES^+^) for C_4_H_8_F_2_O_2_Na (M+Na)^+^: Calcd 149.0385; Measured 149.0384.

### *anti*-4-*tert*-Butyldimethylsilanyloxy-2,3-difluorobutan-1-ol (**8**)


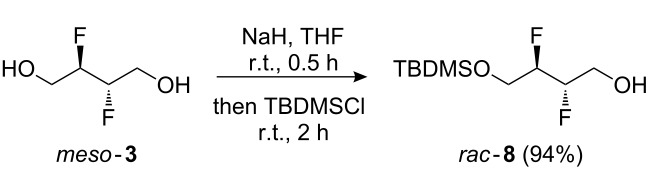


NaH (60% dispersion in mineral oil; 1.40 g, 34.9 mmol) was added to a solution of diol **3** (4.0 g, 31.7 mmol) in THF (64 mL) and the mixture stirred at r.t. for 30 min. TBDMSCl (5.26 g, 34.9 mmol) was then added and the solution stirred at r.t. for a further 2 h. The reaction mixture was quenched with H_2_O (150 mL) and extracted with Et_2_O (200 mL × 3). The combined organic layers were dried over MgSO_4_, filtered and concentrated in vacuo. The crude product was purified by column chromatography (neat petroleum ether, then acetone/petroleum ether 10%) to afford silyl ether **8** as a colourless oil (7.14 g, 94%). IR *ν*_max_ (cm^−1^) 3354 br, 2954 m, 2930 m, 2858 m, 1254 s, 1055 s; ^1^H NMR (400 MHz, CDCl_3_) 4.84–4.58 (2H, m, CHF × 2), 4.03–3.76 (4H, m, CH_2_O × 2), 2.47 (1H, br, OH), 0.91 (9H, s, SiC(CH_3_)_3_), 0.09 (6H, s, SiCH_3_ × 2) ppm; ^1^H{^19^F} NMR (400 MHz, CDCl_3_) 4.77 (1H, ddd, *J* = 6.0, 5.0, 3.0 Hz, CHF), 4.69 (1H, dt, *J* = 6.1, 3.5 Hz, CHF) ppm; ^13^C NMR (100 MHz, CDCl_3_) 90.8 (dd, *J* = 170.5, 21.0 Hz, CHF), 90.5 (dd, *J* = 178.5, 30.5 Hz, CHF), 61.7 (dd, *J* = 21.5, 5.0 Hz, CH_2_O), 61.3 (dd, *J* = 21.5, 5.0 Hz, CH_2_O), 25.9 (SiC(CH_3_)_3_), 18.4 (SiC), −5.38 (CH_3_), −5.43 (CH_3_) ppm; ^19^F NMR (376.5 MHz, CDCl_3_) −201.6 (d, *J* = 13.0 Hz), −201.9 (d, *J* = 13.0 Hz) ppm; ES^+^ *m*/*z* (%) 263 ((M+Na)^+^, 100); HRMS (ES^+^) for C_10_H_22_F_2_O_2_SiNa (M+Na)^+^: Calcd 263.1249; Measured 263.1256.

### *anti*-4-*tert*-Butyldimethylsilanyloxy-2,3-difluorobutyl methanesulfonate (**11**)


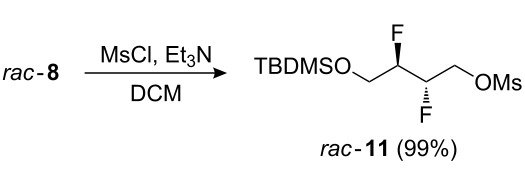


MsCl (3.39 mL, 43.8 mmol) was added to a mixture of alcohol **8** (7.0 g, 29.2 mmol) and Et_3_N (6.6 mL, 46.7 mmol) in DCM (64 mL) and the mixture stirred at r.t. for 2 h. The reaction mixture was cooled to 0 °C, filtered, washed with cold Et_2_O/petroleum ether 1:1 and concentrated in vacuo. The crude product was purified by column chromatography (EtOAc/petroleum ether 15:85) to afford mesylate **11** as a colourless oil (9.29 g, 99%). [TLC monitoring should be performed using DCM/petroleum ether 6:4 until the complete consumption of the starting material, which has the same *R*_f_ value as the product when eluted with EtOAc/petroleum ether.] IR *ν*_max_ (cm^−1^) 2955 m, 2931 m, 2858 m, 1473 w, 1360 s, 1256 m, 1178 s, 836 vs; ^1^H NMR (400 MHz, CDCl_3_) 4.98 (1H, ddtd, *J* = 46.9, 10.1, 6.6, 2.0 Hz, CHCH_2_OS), 4.68 (1H, dddt, *J* = 46.0, 9.6, 6.6, 3.3 Hz, CHCH_2_OSi), 4.62 (1H, ddt, *J* = 26.8, 12.1, 2.0 Hz, CH_a_H_b_OS), 4.49 (1H, dddd, *J* = 25.3, 12.1, 6.1, 2.0 Hz, CH_a_H_b_OS), 3.98 (1H, dddd, *J* = 18.5, 12.5, 3.5, 2.5 Hz, CH_a_H_b_OSi), 3.87 (1H, dddd, *J* = 30.5, 12.5, 3.5, 2.5 Hz, CH_a_H_b_OSi), 3.06 (3H, s, SCH_3_), 0.91 (9H, s, SiC(CH_3_)_3_), 0.09 (6H, s, SiCH_3_ × 2) ppm; ^1^H{^19^F} NMR (400 MHz, CDCl_3_) 4.98 (1H, td, *J* = 6.1, 2.0 Hz, CHCH_2_OS), 4.68 (1H, dt, *J* = 6.6, 3.0 Hz, CHCH_2_OSi) ppm; ^13^C NMR (100 MHz, CDCl_3_) 90.2 (dd, *J* = 176.5, 27.0 Hz, CHCH_2_OSi), 87.5 (dd, *J* = 177.0, 27.5 Hz, CHCH_2_OS), 67.8 (dd, *J* = 21.0, 6.0 Hz, CH_2_OS), 61.3 (dd, *J* = 21.5, 4.5 Hz, CH_2_OSi), 37.7 (SCH_3_), 25.9 (SiC(CH_3_)_3_), 18.4 (SiC), −5.4 (CH_3_), −5.5 (CH_3_) ppm; ^19^F{^1^H} NMR (282 MHz, CDCl_3_) −198.6 (d, ^3^*J*_F-F_ = 13.5 Hz), −202.0 (d, ^3^*J*_F-F_ = 13.5 Hz) ppm; ES^+^ *m*/*z* (%) 341 ((M+Na)^+^, 10); HRMS (ES^+^) for C_11_H_24_F_2_O_4_SSiNa (M+Na)^+^: Calcd 341.1025; Measured 341.1030.

### *anti*-4-Bromo-2,3-difluoro-1-*tert*-butyldimethylsilanyloxybutane (**12**)


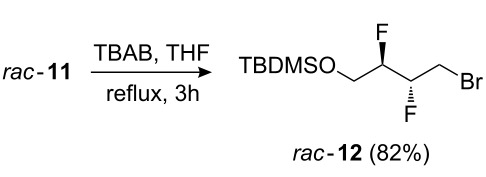


TBAB (9.94 g, 30.8 mmol) was added to a solution of mesylate **11** (8.91 g, 28.0 mmol) in THF (28 mL) and the mixture stirred at reflux for 3 h. The reaction mixture was concentrated in vacuo and the crude product purified by column chromatography (EtOAc/petroleum ether 0% to 25%) to afford bromide **12** as a yellow oil (6.95 g, 82%). IR *ν*_max_ (cm^−1^) 2954 w, 2930 w, 2886 w, 2858 w, 1472 w, 1464 w, 1256 m, 836 vs, 778 s; ^1^H NMR (400 MHz, CDCl_3_) 4.90 (1H, ddtd, *J* = 46.0, 12.0, 6.5, 3.0 Hz, CHF), 4.66 (1H, dddt, *J* = 46.0, 9.0, 6.5, 3.5 Hz, CHF), 3.99 (1H, dddd, *J* = 19.5, 12.0, 3.0, 2.5 Hz, CH_a_H_b_), 3.89 (1H, dddd, *J* = 30.5, 12.5, 4.0, 3.0 Hz, CH_a_H_b_), 3.75 (1H, dddd, *J* = 23.5, 12.0, 3.0, 1.5 Hz, CH_c_H_d_), 3.63 (1H, dddd, *J* = 24.0, 12.0, 6.0, 2.0 Hz, CH_c_H_d_), 0.92 (9H, s, SiC(CH_3_)_3_), 0.10 (6H, s, SiCH_3_ × 2) ppm; ^13^C NMR (100 MHz, CDCl_3_) 91.0 (dd, *J* = 176.5, 27.0 Hz, CHF), 88.1 (dd, *J* = 177.0, 28.0 Hz, CHF), 61.3 (dd, *J* = 21.5, 4.0 Hz, CH_2_OSi), 30.4 (dd, *J* = 22.0, 4.5 Hz, CH_2_Br), 25.8 (SiC(CH_3_)_3_), 18.3 (SiC), −5.5 (CH_3_), −5.6 (CH_3_) ppm; ^19^F NMR (282 MHz, CDCl_3_) −192.6 (d, *J* = 15.0 Hz), −201.3 (d, *J* = 13.0 Hz) ppm; EI *m*/*z* (%) 245 ((M-*t*Bu)^+^, 5), 303 and 305 (1:1, M^+^, 10).

### (*E*)-1-*tert*-Butyldimethylsilanyloxytridec-2-ene (**13**) and (*E*)-1-bromo-4-*tert*-butyldimethylsilanyloxybut-2-ene (**14**)


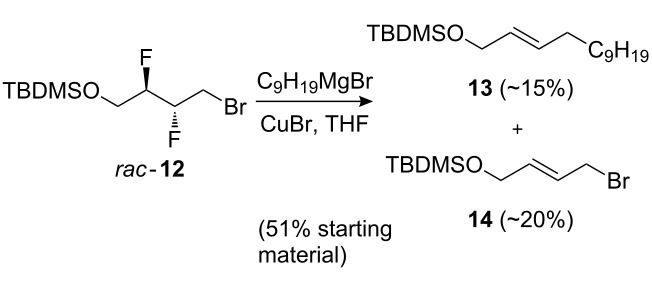


C_9_H_19_MgBr (1.42 mL, 0.6M, solution in Et_2_O, 0.852 mmol) was added to a mixture of CuBr (137 mg, 0.955 mmol) in THF (1.2 mL). The mixture was then transferred to a solution of bromide **12** (140 mg, 0.462 mmol) in THF (1.2 mL) at 0 °C, warmed to r.t. and stirred for 3 h. The reaction mixture was quenched with H_2_O (10 mL) and extracted with Et_2_O (10 mL × 3). The combined organic layers were dried over MgSO_4_, filtered and concentrated in vacuo. The crude product was purified by column chromatography (DCM/petroleum ether 0% to 20%) to afford alkene **13** [54] as a mixture of isomers (1:11) as a yellow oil (24.1 mg, ~15%) and alkene **14** as a yellow oil (26.2 mg, ~20%) along with 72.0 mg (51%) of the starting bromide **12**.

Alkene **13**: IR *ν*_max_ (cm^−1^) 2955 w, 2924 s, 2854 m, 1463 w, 1378 w, 834 s, 774 s; ^1^H NMR ((*E*)-isomer only, 400 MHz, CDCl_3_) 5.64 (1H, dtt, *J* = 15.5, 6.5, 1.5 Hz, CH=CH), 5.53 (1H, dtt, *J* = 15.0, 5.0, 1.0 Hz, CH=CH), 4.13 (2H, dq, *J* = 5.5, 1.5 Hz, CH_2_O), 2.06–2.00 (2H, m, CH_2_), 1.40–1.21 (16H, m, CH_2_ × 8), 0.92 (9H, s, SiC(CH_3_)_3_), 0.93–0.86 (3H, m, CH_3_), 0.08 (6H, s, SiCH_3_ × 2) ppm; ^13^C NMR (100 MHz, CDCl_3_) 131.8 (CH=CH), 129.3 (CH=CH), 64.3 (CH_2_O), 32.4 (CH_2_), 32.0 (CH_2_), 29.8 (CH_2_), 29.7 (CH_2_), 29.5 (CH_2_), 29.4 (CH_2_), 26.2 (SiC(CH_3_)_3_), 22.9 (CH_2_), 18.6 (SiC), 14.3 (CH_3_), −4.9 (SiCH_3_ × 2) ppm; EI *m*/*z* (%) 255.3 ((M-*t*Bu)^+^, 57); HRMS (ES^+^) for C_19_H_40_OSiNa (M+Na)^+^: Calcd 335.2746; Measured 335.2741.

Alkene **14**: Our spectra were in accord with literature copies of the spectra [55]: ^1^H NMR (400 MHz, CDCl_3_) 5.99−5.79 (2H, m, CH=CH), 4.21 (2H, ddd, *J* = 4.0, 2.5, 1.5 Hz, CH_2_), 3.98 (2H, ddd, *J* = 7.5, 2.0, 1.0 Hz, CH_2_), 0.92 (9H, s, SiC(CH_3_)_3_), 0.08 (6H, s, SiCH_3_ × 2) ppm; ^13^C NMR (100 MHz, CDCl_3_) 134.7 (CH=CH), 125.8 (CH=CH), 62.6 (CH_2_O), 32.4 (CH_2_Br), 25.9 (SiC(CH_3_)_3_), 18.4 (SiC), −5.3 (SiCH_3_ × 2); EI *m*/*z* (%) 207 and 209 ((M-*t*Bu)^+^, 31, 1:1); HRMS (EI^+^) for C_6_H_12_O^79^BrSi (M-*t*Bu)^+^: Calcd 206.9835; Measured 206.9841.
